# What will studies of Fulani individuals naturally exposed to malaria teach us about protective immunity to malaria?

**DOI:** 10.1111/sji.12932

**Published:** 2020-09-30

**Authors:** Marita Troye‐Blomberg, Charles Arama, Jaclyn Quin, Ioana Bujila, Ann‐Kristin Östlund Farrants

**Affiliations:** ^1^ Department of Molecular Biosciences The Wenner‐Gren Institute Stockholm University Stockholm Sweden; ^2^ Department of Epidemiology of Parasitic Diseases International Center of Excellence in Research Malaria Research and Training Centre University of Sciences, Technique and Technology of Bamako Bamako Mali; ^3^ CEITEC Masaryk University Brno Czech Republic; ^4^ Department of Microbiology Public Health Agency of Sweden Solna Sweden

**Keywords:** B cells < cells, dendritic cells < cells, inflammation < processes, macrophages < cells, monocytes, parasitic < infections, T cells < cells

## Abstract

There are an estimated over 200 million yearly cases of malaria worldwide. Despite concerted international effort to combat the disease, it still causes approximately half a million deaths every year, the majority of which are young children with *Plasmodium falciparum* infection in sub‐Saharan Africa. Successes are largely attributed to malaria prevention strategies, such as insecticide‐treated mosquito nets and indoor spraying, as well as improved access to existing treatments. One important hurdle to new approaches for the treatment and prevention of malaria is our limited understanding of the biology of *Plasmodium* infection and its complex interaction with the immune system of its human host. Therefore, the elimination of malaria in Africa not only relies on existing tools to reduce malaria burden, but also requires fundamental research to develop innovative approaches. Here, we summarize our discoveries from investigations of ethnic groups of West Africa who have different susceptibility to malaria.

## 1 INTRODUCTION

Malaria is an age‐old scourge of humankind and also has a significant impact on the economic and social development of affected communities.^1^ Malaria is a protozoal blood infection caused by *Plasmodium*, apicomplexan parasites which are transmitted to humans during the bite of a malaria‐infected *Anopheles* mosquito. According to the World Health Organization (WHO), there are over 200 million yearly cases of malaria worldwide, with the heaviest burden of malaria due to *P falciparum* infections in sub‐Saharan Africa.^2^ Malaria is a complex disease, which can manifest on a spectrum from asymptomatic to life‐threatening severe disease, for reasons we do not yet completely understand.^3^

After a period of success in globally reducing the number of malaria cases since the start of the 21st century, the rate of improvement has slowed dramatically over the last five years. This is partly because of increased resistance against existing drugs and insecticides, as well as a lack of new interventions.^2^, ^4^ Effective vaccines for malaria are therefore urgently needed. However, an acknowledged hurdle in the development of effective vaccines is our limited understanding of the biology of *Plasmodium* infection and its complex interactions with the human immune system.

How could one identify immunological mechanisms and correlates of protection to guide next‐generation malaria vaccine development? One could, of course, study samples from clinical trials, where people are protected or not following vaccination. This, however, requires large study groups. Alternatively, one could study samples from controlled human malaria infections, where people are injected with defined numbers of *Plasmodium* sporozoites.^5^ This, however, has obvious practical and ethical limitations. On the other hand, samples from naturally malaria‐exposed sympatric ethnic groups could provide an important key to understanding immunological mechanisms and protection correlates for malaria.

What are sympatric ethnic groups? They are groups of different ancestries that live under similar conditions, including similar socio‐economic factors and in this case similar malaria inoculation rates. Furthermore, they maintain their genetic heritage, for example they do not intermarry between different ethnic groups. For many years, we have been studying such groups living in malaria endemic regions in sub‐Saharan Africa. Particularly, we have studied the Fulani non‐negroid nomadic pastoralists, compared to their negroid sympatric ethnic groups, the Dogon of Mali, or the Mossi and Rimaibé of Burkina Faso.

## 2 THE FULANI ETHNIC GROUP

The Fulani ethnic group has relatively better protection from *P falciparum* malaria than other sympatric ethnic groups. There are over 30 million Fulani distributed across West and Central Africa, particularly in the Sahel region. Fulani populations coincide with regions of very high incidence of *P falciparum* malaria. Since the first report regarding the different responses of Fulani to *P falciparum* infection in 1996,^6^ populations of Fulani from Mali to as far as east Sudan have consistently been reported to have fewer symptomatic cases of malaria, lower *P falciparum* infection rates and lower *P falciparum* densities in infected individuals.^7^, ^8^

Examination of inter‐ethnic genetic differences has shown that the Fulani have a distinct genetic background.^9^, ^10^, ^11^, ^12^ The Fulani are a predominantly Muslim ethnic group and have a preference for intralineage marriages. Genetic studies on the Fulani have shown that the prevalence of already known malaria resistance genes is lower in the Fulani than in other ethnic populations, so this does not account for their better protection.^13^, ^14^

The Fulani have a long history as nomadic herders, seasonally moving with the needs of their livestock. These cultural traditions of the Fulani have resulted in striking lifestyle differences compared to other ethnic groups. For example, Fulani typically have milk cultures, milk products and couscous as their staple foods, while sympatric ethnic groups usually have at least three well‐cooked meals a day.^15^ This has led to speculation that lifestyle factors could also be involved in conferring protection from malaria in the Fulani.^15^, ^16^

Thus, the underlying cause for the lower susceptibility to malaria in the Fulani, genetic or environmental, has been the focus of several studies. But, so far, no conclusive data have been obtained. However, whatever the basis, there is strong evidence that Fulani have different immunological responses to *P falciparum* malaria.

## 3 IMMUNE RESPONSES TO PLASMODIUM INFECTION IN THE FULANI COMPARED TO OTHER SYMPATRIC ETHNIC GROUPS

Studies examining specific characteristics of the immune response to malaria in the Fulani have established, despite similar exposure to the parasite, a number of differences from other sympatric ethnic groups. Fulani are more responsive to *P falciparum* antigens, with higher levels of *P falciparum*‐specific IgG, IgM and IgE antibodies^6^, ^17^, ^18^, ^19^, ^20^, ^21^, ^22^, ^23^, ^24^; Fulani have more activated memory B cells and plasma cells^25^; Fulani have less activated and fewer circulating regulatory T cells^26^, ^27^; Fulani have higher levels of cytokines and chemokines, with higher ratios of pro‐inflammatory to anti‐inflammatory cytokines^20^, ^27^, ^28^, ^29^; and Fulani have increased activation of dendritic cells correlating with their lower frequency in the circulating blood.^29^ Collectively, this suggests a model in which Fulani have more effective innate immune responses to *P falciparum* infection, driving more effective adaptive immunity.^7^ These studies, together with studies of protection from *P falciparum* malaria in other ethnic groups,^30^, ^31^ suggest that early inflammatory innate immune responses can contribute to immunity as well as reduce risk of clinical malaria. However, no direct associations have been established in the Fulani between these differences and the reduced parasite rates or clinical episodes. Thus, the contribution of differences in immune responses in the Fulani regarding the protection against malaria is unclear, as is their underlying cause.^7^

## 4 EPIGENETIC MECHANISMS IN IMMUNITY

**Figure 1:**
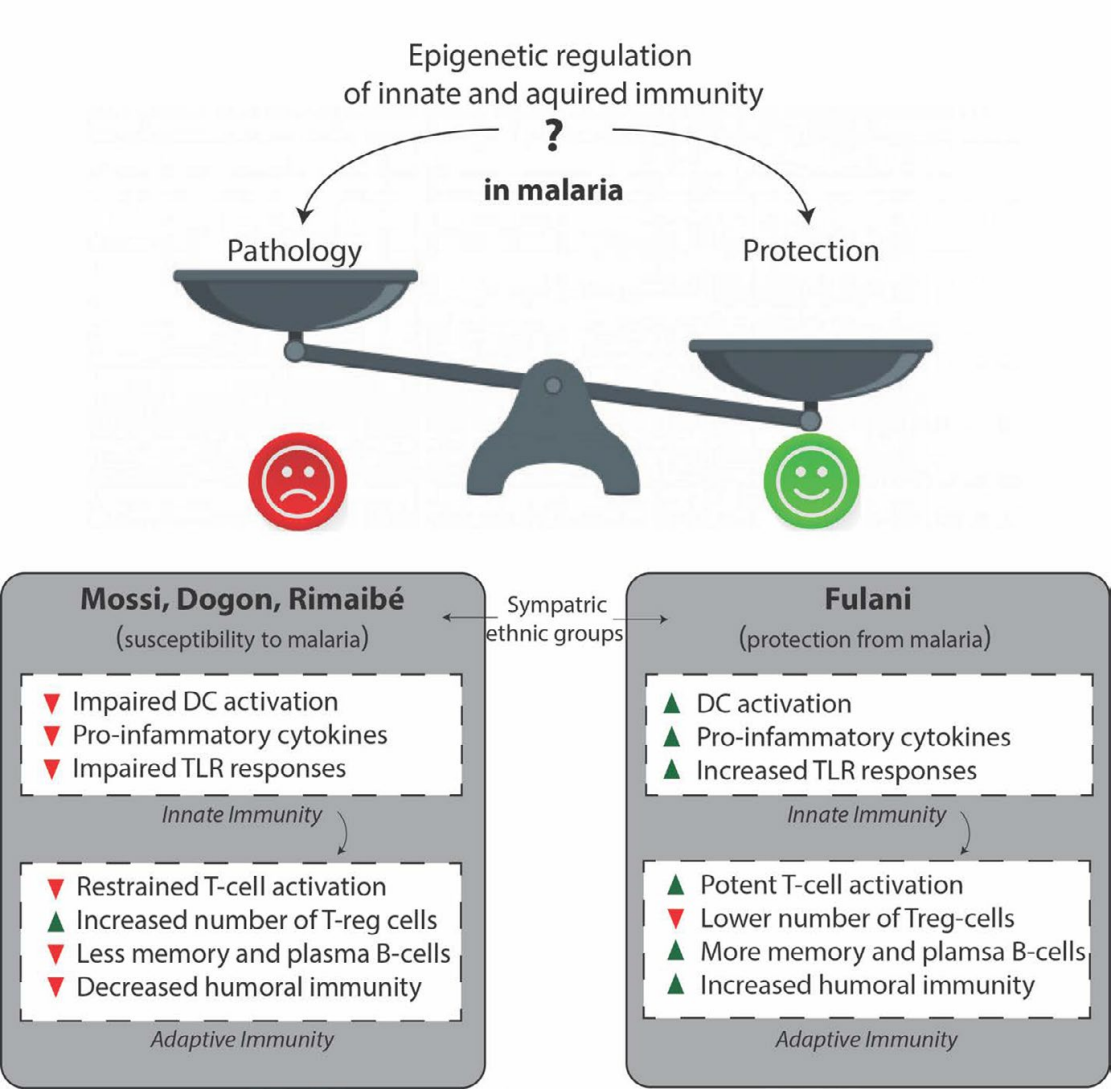
Do epigenetic changes in response to malaria infection contribute to pathology or protection from the disease? Epigenetic changes underlie the development, differentiation and activation of immune cells. We and others have shown that *P falciparum* infection can induce epigenetic changes in innate immune cells: on the one hand, they can contribute to *P falciparum* suppression of innate immune cell function; on the other hand, they can regulate innate immune memory responses. High levels of proinflammatory innate immune responses in malaria could conceivably contribute to either pathology or protection from the disease. In the Fulani ethnic group, who are protected from malaria, we have observed heightened activation of innate immune responses, similar to ‘trained innate immunity’. This suggests that epigenetic activation, and not epigenetic suppression, of innate immunity is protective against malaria

Epigenetic changes underlie the development, differentiation and activation of immune cells, which are regulated by precise spatial and temporal control of gene expression.^32^, ^33^ The major chromatin changes in immune cells occur by DNA methylation and histone modifications, but also ATP‐dependent chromatin remodelling. The DNA methylation pattern primarily changes during haematopoiesis to alter gene expression patterns,^34^ while acetylation and methylation of histones contribute to changes in gene expression also during differentiation and activation of immune cells.^35^, ^36^, ^37^ The epigenetic modification of chromatin is precisely regulated via histone‐modifying enzymes and chromatin remodelling enzymes acting on specific loci, resulting in specific changes in gene expression, chromatin organization and other DNA regulatory processes. Collectively, these mechanisms regulate the expression of key genes that control both the innate and adaptive immune responses (Figure 1).

## 5 INVESTIGATING EPIGENETIC MECHANISMS IN PATHOLOGY OF MALARIA

Attempts to link the better protection from *P falciparum* infection in the Fulani to genetic differences have not been successful. Several nucleotide polymorphisms (SNPs) have been identified in immune genes but none of these were associated with the better protection seen in the Fulani.^38^, ^39^, ^40^ This led us to hypothesize that epigenetic mechanisms together with transcriptional factors or transcription regulators might be involved in shaping the protective immune response to malaria in the Fulani.

For this purpose, we performed genome‐wide transcriptome and DNA‐methylome analysis in CD14+ (monocytes) and CD14‐ (predominantly lymphocytes) from the same individuals, either uninfected or infected with *P falciparum* belonging to the Fulani, or the Mossi sympatric ethnic group, living in the same area and thus exposed to the same level of the parasite. Our results show that Fulani monocytes, specifically, were more transcriptionally reactive to *P falciparum* infection. This was not related to differences in DNA methylation. Rather, several genes involved in chromatin remodelling and epigenetic regulation of gene expression in immune cell lineages are differently expressed, suggesting that the underlying cause is a change in epigenetic regulation in these innate immune cells.^41^

Currently, there are very few studies directly addressing the role of epigenetics in the response to malaria.^42^ However, a number of recent reports indicate that epigenetic changes in innate immune cells may be important for protection from or susceptibility to the disease. On the one hand, epigenetic mechanisms control activation of innate immune memory responses, which can be induced by *P falciparum*. On the other hand, epigenetic mechanisms may contribute to the suppression of host immune responses imposed by *P falciparum*. We are investigating both of these scenarios.

### 5.1 Suppression of host immune responses by *P falciparum*

The malaria parasite has a number of strategies to evade the immune system, and one such method is that *P falciparum* infection can interfere with the functions of innate immune cells.^43^ Specifically, uptake of infected red blood cells or natural hemozoin (nHz), an immunostimulatory product of parasite digestion of haemoglobin, can reduce phagocytic and other functions of these cells, or induce apoptosis.^44^ We asked whether epigenetic regulation, which establishes relatively stable long‐term changes in gene expression, could underlie this immunosuppression.

We have recently shown that monocyte‐derived dendritic cells (DCs) exposed to nHz only partially matured, as indicated by production of high levels of the inflammatory chemokine MCP1, secreted by immature dendritic cells, and a sustained expression of the inflammatory chemokine receptor CCR5, together with an increased expression of maturity markers, such as major histocompatibility complex (MHC) class II and CD86.^45^ Importantly, the presence of nHz could also impair DC maturation in response to treatment with a potent activator of DCs, LPS. We observed that nHz inhibited two hallmarks of DC activation, the loss of podosomes and the expression of CD83. Thus, nHZ is a potent modulator of DC responses.

To elucidate in more detail how nHz affected the DC maturation process, we investigated the binding of transcription factors as well as histone modifications at the promotor region of these genes important in DC maturation. We choose to study the binding of factors downstream of TLR signalling, including NF‐κB and interferon regulatory factors (IRF), as well as the chromatin remodelling factor Brahma‐related gene‐1 (BRG1) in the SWI/SNF complex, which is involved in gene activation. We could not detect any recruitment of NF‐κB subunits (p105/p50 or p65) or IRF3, neither at the transcriptional start site (TSS) nor at the NF‐κB sites following exposure to nHz, in contrast to what usually is seen following stimulation with LPS. These findings suggest an inability of nHz‐exposed DC to recruit certain transcription factors to the promoter regions of genes important for the maturation process. In addition, BRG1 was absent from the promoter region of these genes after nHz exposure. Based on this, we suggest that the necessary remodelling events are also hampered after nHZ exposure. We then investigated the enrichment of various histone modifications, both activating and silencing, at the promoter regions. Our data show that nHz did not enrich for activating histone modifications at these genes. On the contrary, we observed a possible enrichment for the silencing mark H3K27me3 at the TSS of CD83 following nHZ exposure. Thus, it is tempting to speculate that nHZ has the capacity to actively inhibit recruitment of certain transcription factors to the promoter region of genes important in DC maturation via epigenetic mechanisms. In summary, our observations might help to shed light on the molecular mechanisms and stability of suppression of innate immune cell functions by *P falciparum*.

### 5.2 Innate immune memory responses to *P falciparum*

The innate immune system can develop short‐term memory, where a previous challenge results in increased (‘trained’) or decreased (‘tolerized’) response to a second later challenge.^36^ The main mechanism that establishes these states is epigenetic reprogramming at specific genes, which results in innate immune cells being more or less capable of producing inflammatory cytokines, and/or phagocytizing and killing micro‐organisms. In malaria, there is evidence that *Plasmodium* can induce either trained or tolerized innate immune responses. Hyper‐responsiveness has been reported in peripheral blood mononuclear cells (PBMCs) from patients with malaria and correlated with lower rates of reinfection.^46^, ^47^, ^48^ The increased pro‐inflammatory response was associated with activating epigenetic modifications.^49^ On the other hand, depressed responses can occur following multiple malaria infections, with the parasite burden that causes symptomatic infections increasing over time in individuals in endemic areas.^50^, ^51^

High levels of pro‐inflammatory innate immune responses in malaria could be either pathological or protective. Severe pathophysiological events during malaria infection involve erythrocyte destruction and ineffective erythropoiesis, adhesion of *P falciparum‐*infected red blood cells to capillary veins of vital host organs and excessive production and release of pro‐inflammatory cytokines.^52^ Therefore, in escaping host immunity the parasite may prevent the symptoms of severe malaria infection. Or conversely, this could result in far higher levels of parasite and uncontrolled disease. Thus, understanding the mechanisms associated with protection in the Fulani may shed light into how this complex host‐parasite interaction affects the pathology of the disease.

In our genome‐wide study of the Fulani, we observed that the differently expressed genes in monocytes were enriched in immune response (including NF‐κB regulation, IRF3 and IRF7, and the inflammasome subunits), metabolism, RNA metabolism, and chromatin and transcriptional regulation. Importantly, these differences were not observed in the Mossi individuals, nor were significant differences observed in the non‐monocyte cell fraction in Fulani and Mossi individuals. The Fulani had increased levels of pro‐inflammatory cytokines when uninfected (IFNγ, IL‐6), as well as increased levels of IL‐1β and IL‐18 upon infection, indicative of a higher inflammasome activity.^41^ A number of candidate pathways were identified that could contribute to this heightened innate immune response, including genome‐wide regulation of non‐coding RNA and levels of A‐to‐I editing of RNA. Taken together, the heightened response observed in the monocytes of the Fulani shows distinct similarities to the phenomenon of trained innate immune memory, as exemplified by an enhanced transcriptional response in trained cells, which underlies an increased pro‐inflammatory response and other protective immune responses.^53^ However, whether this occurs as a result of training by *P falciparum*, or due to other mechanisms, is still unclear. For example, epigenetic differences that mediate immune responses may be established in response to environmental factors, or as a result of escape from the suppression of host immune response imposed by *P falciparum*.

## 6 CONCLUSIONS

Our studies in the Fulani have identified differences in innate immune responses to malaria. Cells of the innate immune system, such as monocytes, macrophages and dendritic cells, can develop epigenetic states that provide ‘immune tolerance’ or ‘trained immunity’. Recent work has provided support for both suppressive and activating epigenetic changes occurring following malaria infection in different contexts. However, it is so far unclear how this complex interaction affects the pathology of the disease. Our results show that the Fulani, in comparison with the sympatric Dogon and Mossi ethnic groups, exhibit immune responses to the *Plasmodium* parasite similar to ‘trained immunity’. We observe more reactive innate immune cells, with expression of genes involved in innate immunity, metabolism and chromatin remodelling, as well as higher levels of pro‐inflammatory cytokines. However, we do not yet know when or how the changes underlying the transcriptional response seen in the Fulani are established. Our further studies performing genome‐wide analysis of chromatin states in the Fulani will shed light on these questions. Nevertheless, our data indicate that monocytes of uninfected Fulani are already set in a ‘high alert’ state, enabling a stronger reaction upon *P falciparum* infection, suggesting this phenomenon is correlated with protection from malaria. These findings may provide a key to new/improved vaccine candidates and anti‐malarial drugs.

## CONFLICT OF INTEREST

The authors agree on the contents of the manuscript and declare that the research was conducted in the absence of any commercial or financial relationship that could be considered as a potential conflict of interest.

## AUTHOR CONTRIBUTIONS

MTB conceived and outlined this review manuscript. AÖF, CA, JQ and IB assisted in writing and editing this manuscript and approved the final version.
